# Consecutive Image Acquisition without Anomalies

**DOI:** 10.3390/s24206608

**Published:** 2024-10-14

**Authors:** Angel Mur, Patrice Galaup, Etienne Dedic, Dominique Henry, Hervé Aubert

**Affiliations:** 1 Ovalie Innovation, 32000 Auch, France; patrice.galaup@gmail.com (P.G.); dhenry@laas.fr (D.H.); 2 LAAS-MINC-Equipe MIcrop et Nanosystèmes pour les Communications sans fil, 31400 Toulouse, France; ededic@laas.fr (E.D.); aubert@laas.fr (H.A.)

**Keywords:** anomaly detection, anomaly correction, optical measurement, dynamic time warping distance, Wasserstein distance

## Abstract

An image is a visual representation that can be used to obtain information. A camera on a moving vector (e.g., on a rover, drone, quad, etc.) may acquire images along a controlled trajectory. The maximum visual information is captured during a fixed acquisition time when consecutive images do not overlap and have no space (or gap) between them. The images acquisition is said to be *anomalous* when two consecutive images overlap (overlap anomaly) or have a gap between them (gap anomaly). In this article, we report a new algorithm, named *OVERGAP*, that remove these two types of anomalies when consecutive images are obtained from an on-board camera on a moving vector. Anomaly detection and correction use here both the Dynamic Time Warping distance and Wasserstein distance. The proposed algorithm produces consecutive, anomaly-free images with the desired size that can conveniently be used in a machine learning process (mainly Deep Learning) to create a prediction model for a feature of interest.

## 1. Introduction

A camera installed on a mobile vector, such as a rover or a drone, may be used to acquire images along a controlled trajectory (see, e.g., [1,2,3,4]). More specifically, we can think of a camera on a rover that moves between the rows of a vineyard to capture images of the plants (see, e.g., [2]). These acquired images can be used to build models to predict diseases, yield, etc. [1,5].

**Figure 1:**
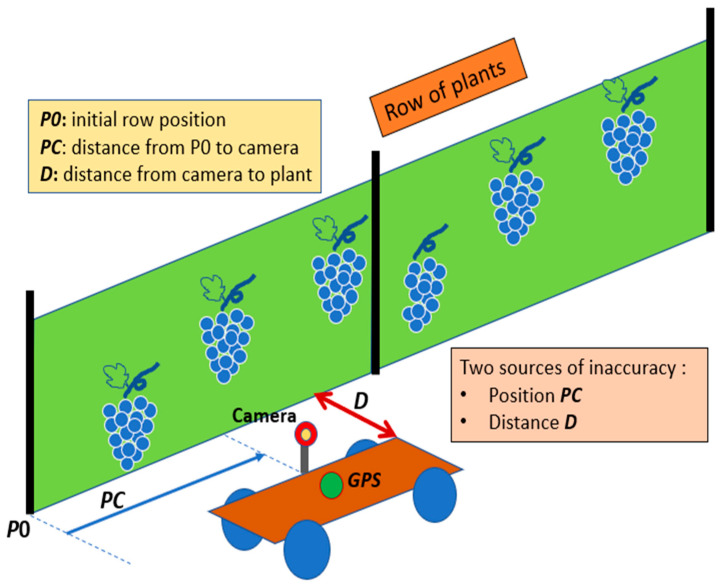
Context of the image acquisition system using an on-board camera on a moving vector (rover).

In the illustration of Figure 1, the mobile vector is equipped with a camera to acquire sequences of images and a Global Positioning System (*GPS*) receiver [6] to estimate the corresponding camera positions over time. As the vector progresses along the furrows, the camera captures information through a set of consecutive frames.

The vector may not follow during time a path which is strictly parallel to the scene, since the area could be non-uniform or/and the vector could be driven manually. As the distance between the camera and the row of plants is not fixed during the rover displacement, the acquired images overlap or have undesirable gaps between them. Moreover, from using an on-board *GPS* receiver, the position of the camera can be estimated with unavoidable uncertainties. A global navigation satellite system provides actually geolocation and time information to a *GPS* receiver anywhere on or near the Earth where there is an unobstructed line of sight to four or more *GPS* satellites [6]. The geolocation precision can be a few meters with a standard *GPS* and can reach a few cm using Differential *GPS* (*DGPS*) [7].

When consecutive images do not overlap and have no space (or gap) between them, the maximum visual information is captured by the image acquisition system during a fixed acquisition time. The images acquisition is said to be *anomalous* when two consecutive images overlap (overlap anomaly [4]) or have a gap between them (gap anomaly).

Image stitching is the process of combining multiple images with overlapping fields of view to produce a single larger image. In image stitching, multiple image pairs are successively registered relative to each other to form a panorama. In [4], the authors report an automated prediction of vineyard yield from image acquisition in which two consecutive images contain a repeated vine segment and, consequently, only the overlap anomaly is considered. Consecutive images were matched using the ‘Auto Blend’ tool provided by Adobe Photoshop (Adobe Systems Incorporated; San José, 179 CA, USA) [8] and the correction of the overlap anomaly is performed offline. However, ref. [4] does not explain how the overlap is kept between consecutive images, and additionally does not consider gap anomalies. A comparison of different stitching algorithms (*SIFT*, *SURF*, *KAZE*, *AKAZE*, *ORB* and *BRISK*) is presented in [9,10]. Ref. [11] explains a new method to solve the perspective deformation and misalignment in image stitching. Ref [12] presents an enhanced *SIFT* method designed for the stitching of underwater images characterized by smeared feature contours. Ref. [13] presents a method for automatic image stitching of agriculture areas based on utilizing an unmanned aerial vehicle using *SURF*.

In this article, we report a new real-time algorithm, named *OVERGAP*, to acquire consecutive images without overlap and gap anomalies. This algorithm offers a different solution from the stitching algorithms mentioned above. These stitching methods need to detect some matched features between consecutive images. However, when working with vineyard images, they have difficulties in detecting a sufficient number of matched features. *OVERGAP* works differently and detects anomalies that these stitching methods are not able to correct. We have worked here exclusively with images in which the anomalies have been simulated.

The algorithm allows for collecting all the visual information through a set of consecutive images sharing the same dimension. This set is useful for building prediction models of a feature of interest (e.g., the vineyard yield of a plot) based on supervised learning.

The *OVERGAP* algorithm constitutes a new approach to anomaly detection and correction. It allows for detecting anomalies during the acquisition of consecutive images and correcting them. The algorithm fills in missing information from a gap anomaly and removes redundant information from an overlap anomaly. It could act prior to the use of a prediction model. This approach contrasts with other works where learning techniques are used to create a model with the sole purpose of detecting and correcting anomalies. For example, in [14] anomalies are detected in an unsupervised manner and in [15] they are detected and corrected using a model obtained in a supervised manner. The anomalies in [14] and [15] are different from those detected and corrected by the proposed *OVERGAP* algorithm.

The article is organized as follows: in Section 2, we explain in detail the inputs used by the *OVERGAP* algorithm and in Section 3, the algorithm is described. Section 4 and Section 5 report the application of *OVERGAP* to detect and correct anomalies. Section 6 analyzes other image stitching methods. Section 7 discusses the methodology. Finally, Section 8 concludes the article.

## 2. Background

In this section, some concepts useful for the *OVERGAP* algorithm are reported.

### 2.1. Key Features of Cameras for the OVERGAP Algorithm

**Figure 2:**
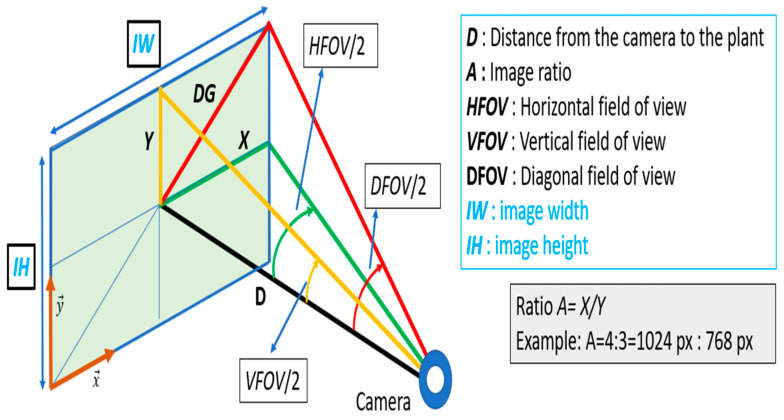
Parameters of the camera and size of the captured surface appearing in the image.

Figure 2 indicates the three characteristic parameters of a camera in the visible domain, namely the image resolution (*IR*) in pixels, the image ratio (*A*) and the field of view (*FOV*). The provided field of view can be horizontal (*HFOV*), vertical (*VFOV*) or diagonal (*DFOV*). From these parameters, a camera located at a distance *D* from the scene will produce an image where the dimensions of the captured surface appearing in the image can be estimated.

The variable *X = IW*/2 is calculated from Equation (1) depending on the type of *FOV*:

(1) $$\;X=\frac{D\times \tan\left(\frac{DFOV}{2}\right)}{\sqrt{\left(1+\frac{1}{A^{2}}\right)}}=D\times \tan\left(\frac{HFOV}{2}\right)=A\times D\times \tan\left(\frac{VFOV}{2}\right)$$

The parameters *IW* and *IH* can be derived from Equation (1), as follows:

(2) $$IW=2\times X\;\;and\;IW=2\times \frac{X}{A}$$

For example, a Microsoft LifeCamStudio camera [16] has a FullHD resolution of 1920 × 1080 px and a *DFOV* of 75°. If we consider that *D* = 1 m, we obtain *IW* = 1.33 m and *IH* = 0.75 m.

### 2.2. The Dynamic Time Warping Distance

In time series analysis, Dynamic Time Warping (*DTW*) is a standard algorithm used for estimating the degree of similarity between two temporal sequences, which may vary in speed (see, e.g., [17,18,19]). For example, similarities could be detected in the gait of two people walking at different speeds. The main idea of the *DTW* technique is to estimate the distance from the matching of similar elements between time series. This distance will be used in the *OVERGAP* algorithm to detect overlap and gap anomalies in consecutive images. This distance is effective for comparing columns of different information. As will be detailed in Section 3.2 and Section 3.3, the *OVERGAP* algorithm derives from this distance which column in the image is most similar to a specific column in another image.

### 2.3. The Wasserstein Distance

The Wasserstein Distance (*WD*) is a similarity metric between two probability distributions (see, e.g., [20,21]). This distance is used here by *OVERGAP* algorithm to correct eventual anomalies during the acquisition of consecutive images. As shown in Section 3.2 and Section 3.3, it is mainly used to correct the distortion of the image during the displacement of the moving vector. *WD* is effective for comparing columns of similar information where the columns have a different scaling. The *OVERGAP* algorithm detects with *WD* how different the scaling is in order to resize an image. For each column a histogram is obtained. This depends on the number of bins selected. The *Freedman Diaconis Estimator* [22] has been used here to obtain the number of bins. This estimator is robust with respect to outliers and considers the variability and size of the data.

## 3. *OVERGAP* Algorithm

The *OVERGAP* algorithm is composed of three steps: (1) image acquisition, (2) anomaly detection and (3) anomaly correction. This section describes how often an image should be acquired. Subsequently, the detection and correction of (overlap and gap) anomalies are explained. At the end, we show how *OVERGAP* can work in real time.

### 3.1. Image Acquisition

**Figure 3:**
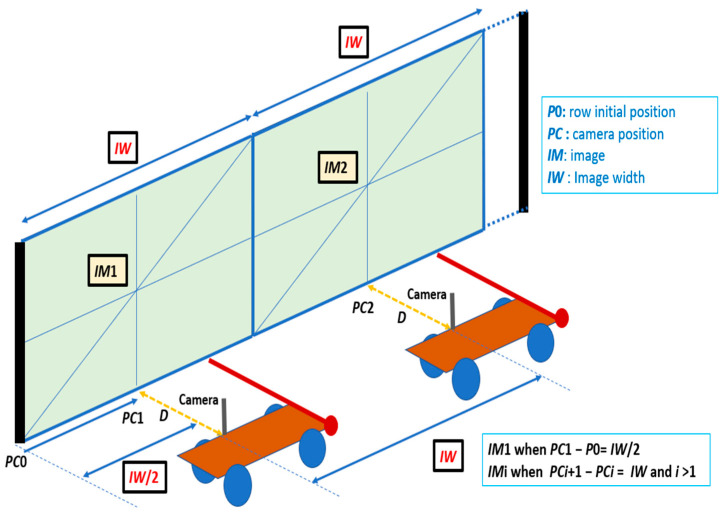
Acquisition process of consecutive images when the distance *D* and the position *PC* are certain (i.e., without errors).

Figure 3 sketches the acquisition process of consecutive images when the distance *D* and the position *PC* of the camera are certain (i.e., not tainted by errors). As mentioned above, the width of the image content spans *IW* m. In this case, all the information can be acquired at a distance *D* without anomalies. It is sufficient to perform a first acquisition when the camera position is at a distance *IW*/2 from the start and, subsequently, every *IW* m.

**Figure 4:**
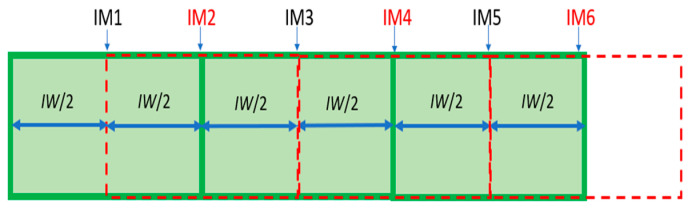
Acquisition of images every *IW*/2 m.

When there are anomalies between consecutive images, image acquisition must be corrected, because more images are necessary in addition to the consecutive images to correct anomalies. For example, in Figure 4, there are six images that have been obtained each time the device progresses by the distance *IW*/2 m. The odd numbered images (*IM*1, *IM*3, *IM*6, …) in green are the main images (*MI*). The even images (*IM*2, *IM*4, *IM*6, …) in red are the secondary images (*SI*). The *OVERGAP* algorithm is applied between each pair of consecutive *MI*s. The even image between these images is used to detect and correct the anomaly.

Acquiring an image every *IW*/2 m is one of the important points of the *OVERGAP* algorithm. The anomaly between consecutive images can be detected and corrected as long as the corresponding even image includes the overlap or gap.

**Figure 5:**
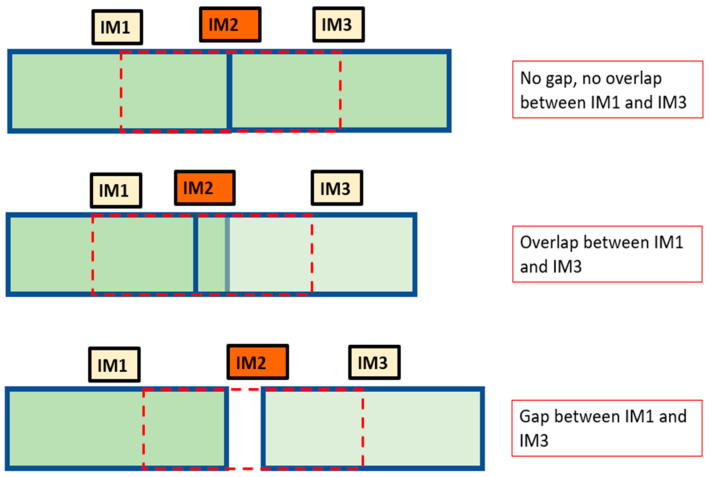
Acquisition of *IM*1 and *IM*3 together with *IM*2. In the case of an anomaly, the overlap or gap must be included in *IM*2.

Figure 5 shows the three possible cases during the acquisition of images *IM*1, *IM*2 and *IM*3:

(1) there is no anomaly between *IM*1 and *IM*3;
(2) there is overlap between *IM*1 and *IM*3 that is included in *IM*2;
(3) there is a gap between *IM*1 and *IM*3 that is included in *IM*2.

### 3.2. Overlap Detection and Correction

**Figure 6:**
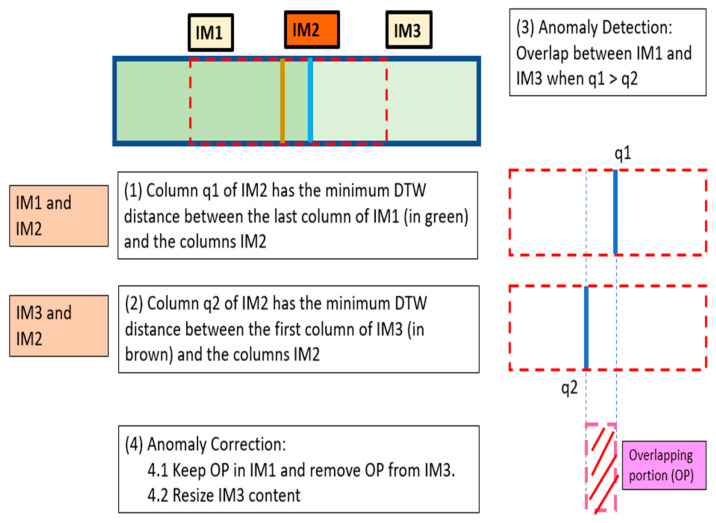
Two consecutive *MI*s (*IM*1, *IM*3) and their corresponding *SI* (*IM*2). Overlap anomaly occurs.

Figure 6 shows two consecutive *MI*s (*IM*1, *IM*3) and their corresponding *SI* (*IM*2). There is an overlap between the *MI*s and, therefore, an overlap anomaly occurs.

To detect anomalies, *OVERGAP* uses the *DTW* distance in order to find the column of *IM*2 that is the intersection with the last column of *IM*1. The position of the column with the minimum distance is *q*1. Likewise, *OVERGAP* looks for the column of *IM*2 that is the intersection with the first column of *IM*3. The position of the column with the minimum distance is *q*2. Since *q*1 > *q*2, an overlap anomaly has been detected and the region between *q*1 and *q*2 of *IM*2 is the Overlapping Portion (*OP*).

*IM*1 does not undergo any modification. *IM*3 will suffer from *OP*’s cropping. However, this cut will be made after analyzing whether it is necessary to resize the content of *IM*3 since in our model the distance *D* may vary.

The last column of *IM*1 (*LC_IM*1) is compared with the column *q*2 of *IM*3 (*C*2_*IM*3) to resize the content of *IM*3. To do this, the histogram of *LC_IM*1 is obtained. Subsequently, different *C*2_*IM*3 columns are obtained by scaling the content of *C*2_*IM*3 in different ways. For example, increasing or decreasing the dimension by n pixels with −*N* <= *n* <= *N* where, for example, *N* = 20. If the result of this scaling is a column with more pixels, then it is cropped to have the same dimension as *LC_IM*1; if the result is a column with fewer pixels, then pixels are added (with a value equal to the closest calculated) to have the same dimension as *LC_IM*1. The histogram is obtained for each of the new columns of *C*2_*IM*3. Subsequently, *WD* is calculated with the histogram of *LC_IM*1. *IM*3 is resized according to the *y* axis in the same way as the *C*2_*IM*3 column that has the minimum *WD* distance. In the end, we obtain *IM*1 and the new image *IM*3 resized without *OP* (*N_IM*3). If required, both can be concatenated and cut again to find the desired size according to the *x* axis. The new *IM*1 (*N_IM*1) will have the desired size. If the size of *N_IM*3 is different, then the algorithm will wait to obtain the next images *IM*4 and *IM*5 to obtain *N_IM*3 with the same size than *N_IM*1 following the same process as *IM*1, *IM*2 and *IM*3.

### 3.3. Gap Detection and Correction

**Figure 7:**
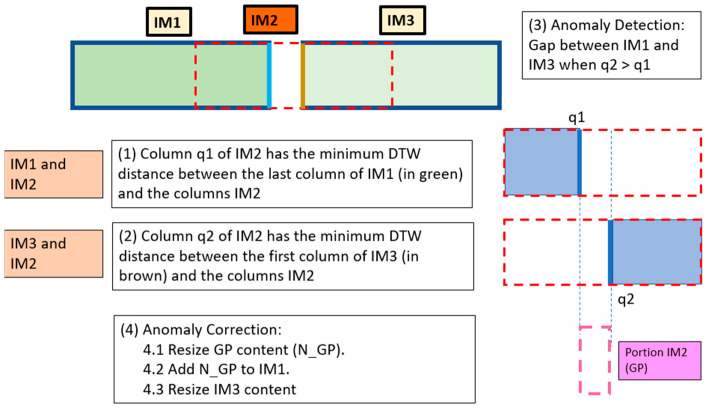
Two consecutive *MI*s (*IM*1, *IM*3) and their corresponding *SI* (*IM*2). Gap anomaly occurs.

Figure 7 shows two consecutive *MI*s (*IM*1, *IM*3) and their corresponding *SI* (*IM*2). There is a gap between the *MI*s and therefore a gap anomaly occurs.

The *OVERGAP* algorithm uses the *DTW* distance to detect *q*1 and *q*2 in a similar way as explained in Section 3.2. However, since *q*1 < *q*2, then a gap anomaly has been detected and the region between *q*1 and *q*2 of *IM*2 is the Gap Portion (*GP*).

Once the gap anomaly is detected, unlike the overlap anomaly, two corrections need to be made according to the *y* axis. The first correction is performed on *GP*. The procedure is similar to that explained in the previous section, although now *LQ_IM*1 is compared with several columns that are formed from the scaling of column *q*1 of *IM*2 (*C*1*_IM*2). The minimum *WD* allows us to select how much *GP* should be resized and, consequently, we derive a new portion *N_GP* where the first column is omitted.

The second correction according to the *y* axis uses the first column of *IM*3 (*FQ_IM*3) and the columns that are formed by scaling the last column of *N_GP* (*LQ_N_GP*). The *IM*3 is resized according to the *y* axis in the same way as the *LQ_N_GP* column that has the minimum *WD* distance. The result is the new image named *N_IM*3.

In the end, images *IM*1, *N_GP* and *N_IM3* are concatenated. The new *IM*1 (*N_IM*1) will have the desired size, while the rest corresponds to *N_IM*3. If the dimension of *N_IM*3 according to the *x* axis is different from the dimension of *N_IM*1, then the algorithm will wait the acquisition of the next images *IM*4 and *IM*5. The new anomaly (if it occurs) is detected and corrected using the images *N_IM*3, *IM*4 and *IM*5. After concatenation, a first cut is made for *N_IM*3 of the same dimension as the dimension of *N_IM*1, while the rest corresponds to *N_IM*5.

### 3.4. The OVERGAP Algorithm in Real-Time

The *OVERGAP* algorithm can work in real-time on the processor of a mobile vector. The mobile device places the camera in the PC0 position. When the device moves, the processor with the help of *DGPS* evaluates the position of the device (*PCi* with *i >* 0) every few milliseconds. Every time the vector travels *IW*/2 m, *OVERGAP* analyzes if there is an anomaly and, if so, corrects it:
*i = 1**if PCi − PCi-1 >= IW/2,*       *IMi is measured*       *if i is odd and >1 (Two consecutive MIs),*          *OVERGAP using IMi-2, IMi-1 and IMi**i = i + 1*

The algorithm can work in real time as long as *OVERGAP* ends before the time the vector needs to travel *IW*/2 m. The *IW* is obtained from the camera parameters. The maximum speed of the vector will be conditioned by *IW* and the processing speed of the processor. The processing speed can also be increased if *OVERGAP* works with images of smaller dimension than those acquired. In this case, the calculation of the *DWT* and *WD* distances is faster.

Suppose that *OVERLAP* works on a processor that performs an image acquisition every *S = IW*/2 (in meters). Once an image *IMi* is obtained, the *OVERGAP* algorithm is applied to *IMi*-2, *IMi*-1 and *IMi*. Suppose that the processor uses a time *T* (in seconds) to apply *OVERGAP*. If we consider that the device moves at a uniform speed, the processor will work in real time if the speed of the mobile V ≤ S/T (in meters/second). Therefore, the solution is not unique.

The *OVERGAP* algorithm using *IMi-2, IMi-1* and *IMi* is summarized as follows:
*Given a set of three consecutive images IMi-2, IMi-1 and IMi:**1. Calculate q1 using IMi-2, IMi-1 and DTW.**2. Calculate q2 using IMi-1 and IMi and DTW.**3. If q1 > q2: overlap anomaly between IMi-2 and IMi.*        *3.1 Anomaly correction: Keep OP in IMi-2 and remove OP from IMi.*
        *3.2 Anomaly correction: Resize IMi content (N_IMi) with the help of WD.*        *3.3 Concatenate IMi-2 and N_IMi and cut to find the desired size according to the x axis.**4. If q1 < q2: gap anomaly between IMi-2 and IMi.*        *4.1 Anomaly correction: Resize GP content (N_GP) with the help of WD.*        *4.2 Anomaly correction: Add N_GP to IMi-2 (N_IMi-2).*
        *4.3 Anomaly correction: Resize IMi content (N_IMi) with the help of WD.*        *4.4 Concatenate N_IMi-2 and N_IMi and cut to find the desired size according to the x axis.**5. If q1 = q2: there is no anomaly.*

## 4. Illustrative Examples

This section shows some simulated examples to visually analyze the efficiency of the *OVERGAP* algorithm in detecting and correcting both overlap and gap anomalies.

### 4.1. Detection and Correction of Overlap Anomaly

**Figure 8:**
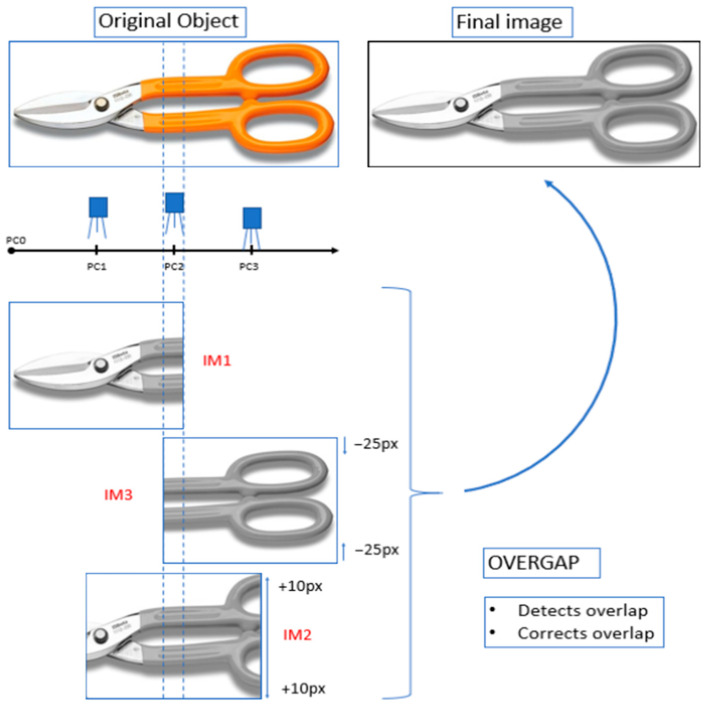
Example of detection and correction of an overlap anomaly: A “camera” obtains images *IM*1, *IM*2 and *IM*3 in positions *PC*1, *PC*2 and *PC*3, respectively. The content of *IM*2 has also been resized in +10 px to simulate an increase in *D.* The content of *IM*3 has been resized by −25 px to simulate a decrease in *D*. The final image is the concatenation of IM1 together with N_IM3.

Figure 8 presents an object that is photographed by a “camera” three times in positions *PC*1, *PC*2 and *PC*3 to obtain images *IM*1, *IM*2 and *IM*3, respectively. Each image has a resolution of 360 × 283 px. There is an overlap anomaly between *IM*1 and *IM*3. In addition, *IM*2 has been resized to simulate a decrease in *D* and *IM*3 has also been resized to simulate an increase in *D*. The *OVERGAP* algorithm detects the anomaly and corrects it. The result is the final image showing the concatenation of *IM*1 together with N_IM3. For the sake of simplicity, the algorithm works with gray images.

### 4.2. Detection and Correction of Gap Anomaly

**Figure 9:**
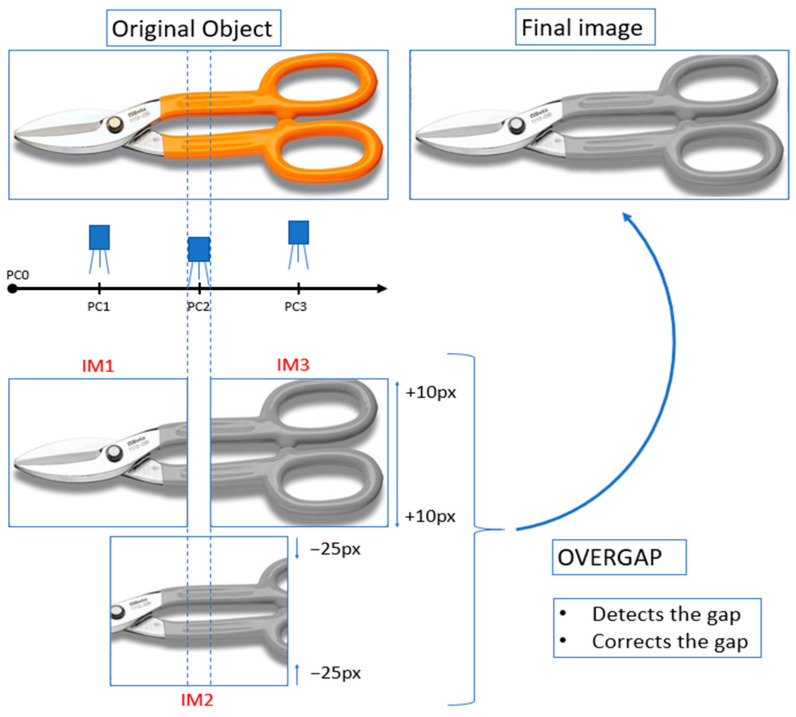
Example of detection and correction of a gap anomaly: A “camera” obtains images *IM*1, *IM*2 and *IM*3 in positions *PC*1, *PC*2 and *PC*3, respectively. The content of *IM*2 has been resized by −25 px to simulate a decrease in *D*. The content of *IM*3 has also been resized in +10 px to simulate an increase in *D.* The final image is the concatenation of *IM*1, *N_GP* and *N_IM3*.

Figure 9 presents an object that is photographed by a “camera” three times in positions *PC*1, *PC*2 and *PC*3 to obtain images *IM*1, *IM*2 and *IM*3, respectively. Each image has a resolution of 320 × 283 px. There is a gap anomaly between *IM*1 and *IM*3. In addition, *IM*2 has been resized to simulate an increase in *D* and *IM*3 has also been resized to simulate a decrease in *D*. The *OVERGAP* algorithm detects the anomaly and corrects it. The result is the final image showing the concatenation of *IM*1, *N_GP* and *N_IM3*. The final image has a slight crop in the top hole. This was expected, as *IM*3 already has a cut due to the decrease in *D* in *PC*3.

## 5. The *OVERGAP* Algorithm in the Context of Precision Agriculture

The *OVERGAP* algorithm uses a camera placed on a mobile vector. In precision agriculture, this camera acquires images of a row of plants.

**Figure 10:**
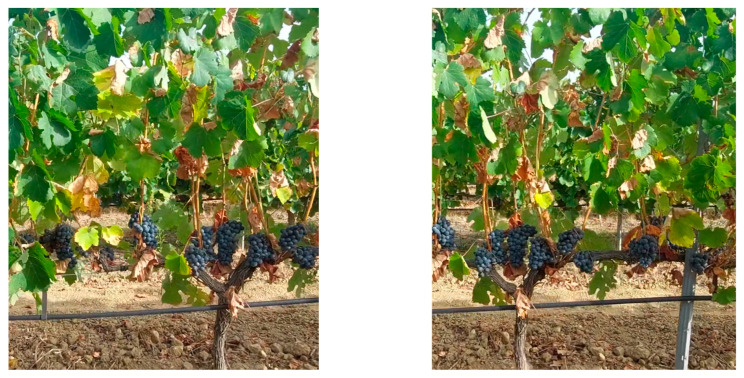
The same plant is observed from two different points of view as the mobile vector advances.

The main problem for *OVERGAP* is that two images taken from different positions of the same plant can present significant differences. Mainly, the leaves are displayed differently. Figure 10 shows two images where the same plant is observed from two different points of view as the mobile vector advances. The leaves look different.

**Figure 11:**
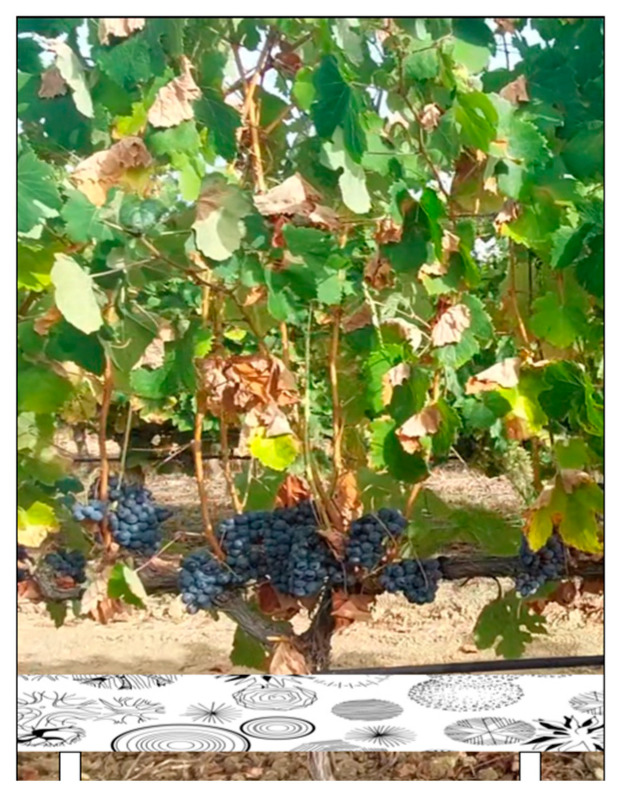
A vine plant with a band of drawings.

**Solution 1:** One solution for OVERGAP to be successfully applied in precision agriculture is to place a strip with different drawings at the base of the row of plants. Figure 11 shows the image of a vine plant with a band of drawings. The camera captures the image of the plant and the strip simultaneously. *OVERGAP* works only with the lower part of the images containing the strip with drawings. The result is applied to the rest of the content of the corresponding images. This solution gives the best results in anomaly correction, since the behavior of the algorithm on the strip image is similar to the scissors example of Section 4.

**Figure 12:**
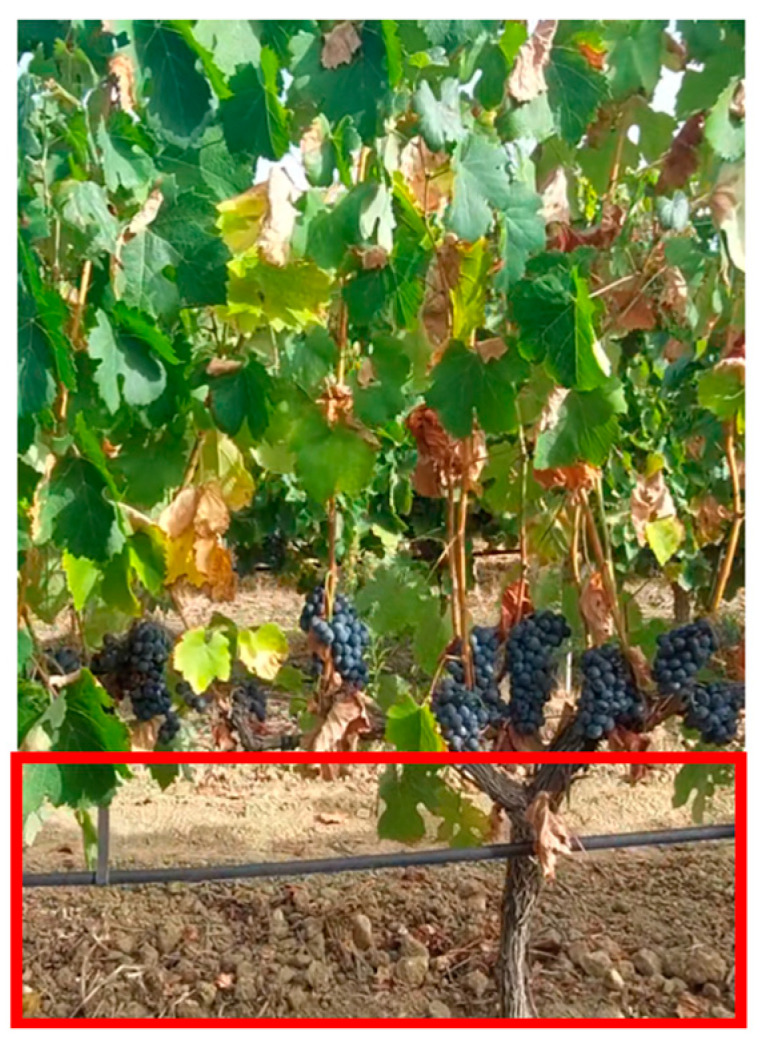
The lower part of the image has hardly any leaves.

**Solution 2:** Another less efficient solution than the previous one would be to work with an area of the image with fewer leaves. In Figure 12, the lower part of the image is highlighted with a red rectangle. The advantage of this approach is that it does not require a strip with drawings.

**Figure 13:**
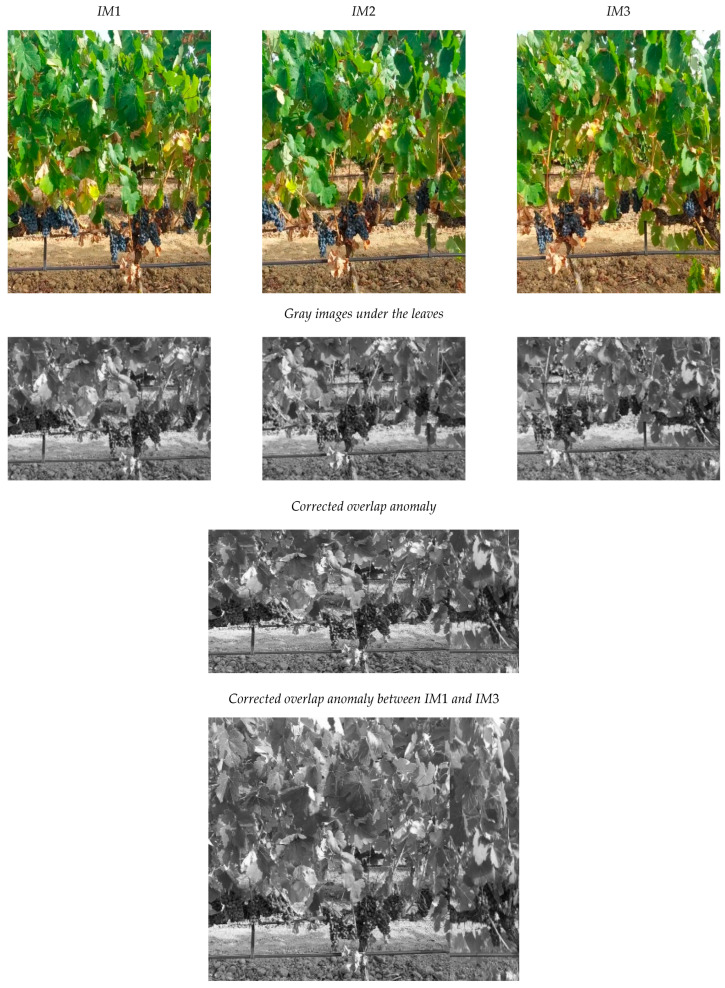
Example of detection and correction of an overlap anomaly using images from a vineyard.

An overlap anomaly has been simulated using a sequence of three consecutive images of vineyard plants. Figure 13 shows the images and the result of correcting this anomaly using the lower area of the images.

**Figure 14:**
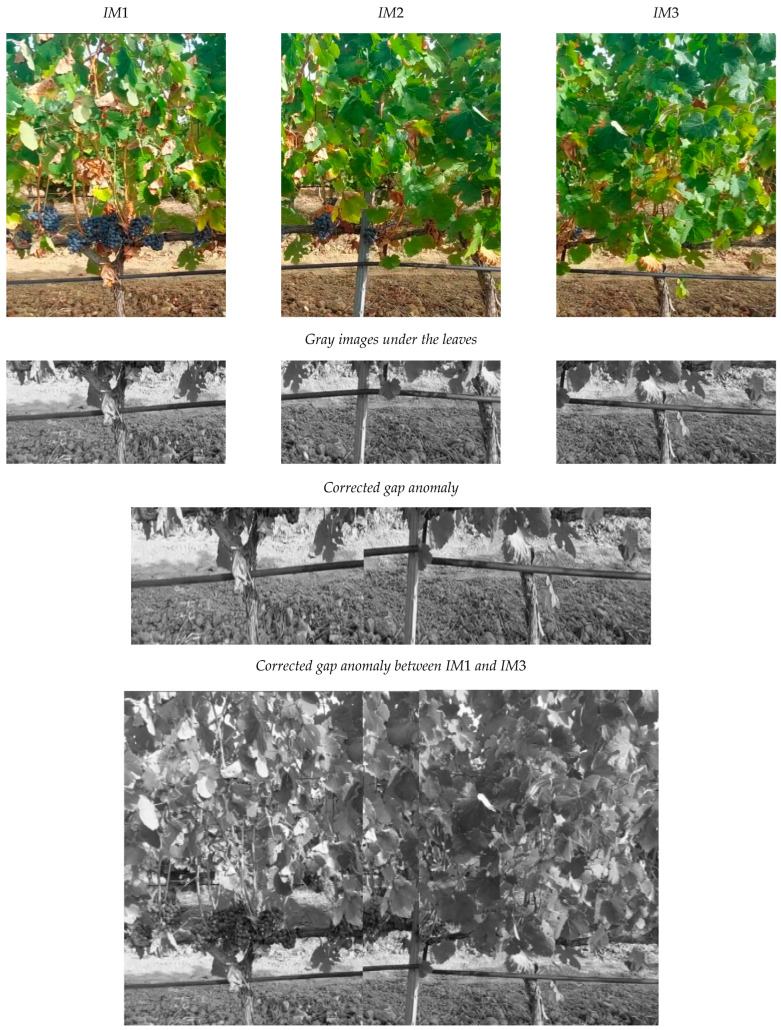
Example of detection and correction of a gap anomaly using images from a vineyard.

A gap anomaly has been simulated using a sequence of three consecutive images of vineyard plants. Figure 14 shows the images and the result of correcting this anomaly using the lower area of the images.

Depending on the development of the plant, the lower part of the image may have more or less leaves. Instead of using a single sub-image, three or more different sub-images can be selected. For each sub-image, the corresponding *q*1 and *q*2 are obtained. An average is then taken to determine the corresponding *OP* or *GP*. An average can also be used to determine the scaling obtained with *WD*.

Scaling corrects images when the mobile vector does not respect the distance to the plant during the trajectory. This is effective with solution 1, since the strip of drawings does not have leaves and is always flat with respect to the camera. On the contrary, with solution 2, scaling is no longer as effective since it is performed from the image without the strip.

The distance from the mobile vector to the plant can be constant and still find differences in the continuity of the images. The ground is not perfectly flat. In our example, it is easy to see that there is no continuity of the irrigation hose even if the distance from the vector to the plant is constant. And this cannot be adequately corrected by scaling. Although scaling is not always useful, the images obtained are still valid for use in a learning algorithm.

## 6. Correction of Anomalies Using Other Image Stitching Methods

In this section, we have tried other image stitching methods (*SIFT*, *SURF*, *KAZE*, *AKAZE*, *ORB* and *BRISK*) to correct the anomalies in Figure 13 and Figure 14.

To create the panorama, those algorithms register successive image pairs *IM*(*n*) and *IM*(*n* − 1) using the following procedure:
Detect and match features between *IM*(*n*) and *IM*(*n* − 1).Estimate the geometric transformation, *T*(*n*), that maps *I*(*n*) to *I*(*n* − 1).Compute the transformation that maps *I*(*n*) into the panorama image as *T*(1)∗*T*(2)∗ …∗*T*(*n* − 1)∗*T*(*n*).

Stitching methods differ mainly in how they determine the matched features between *IM*(*n*) and *IM*(*n* − 1). The effectiveness of image stitching heavily depends on the image quality and the number of matched features.

We have used a Python package [23] to test these methods.

**Figure 15:**
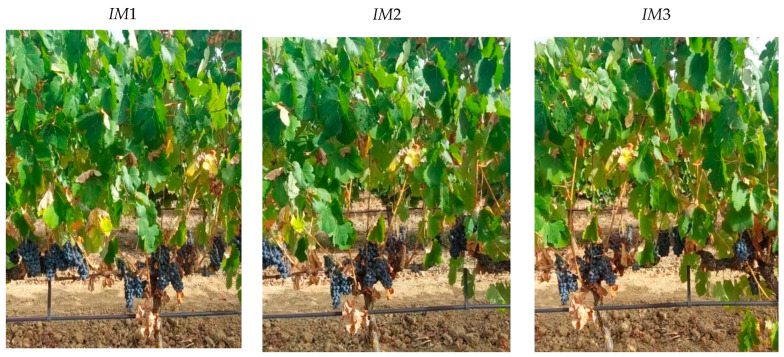

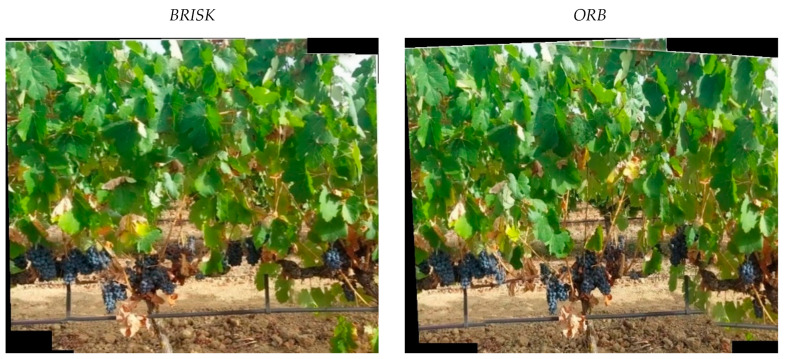
Example of application of the *BRISK* (**left**) and *ORB* (**right**) algorithms for the detection and correction of an overlap anomaly using images of a vineyard.

Regarding the consecutive images in Figure 13 (overlap anomaly), only *BRISK*, *ORB* and *SURF* yielded results. The other detectors could not find matched features between consecutive images. *BRISK* provided the best result. Unlike *OVERGAP*, it left *IM*3 intact and cut *IM*1. The *ORB* result is less accurate and the *SURF* result is worse. Figure 15 shows the *BRISK* and *ORB* panorama.

Regarding the images of Figure 14 (gap anomaly), none of the stitching methods were able to obtain a panorama and, therefore, the gap anomaly has not been corrected.

These methods usually fail when they are unable to detect matched features between consecutive images. To find matched features, these algorithms compare the images using all available information. The vineyard images used are difficult to process because the leaf orientation changes rapidly between consecutive images.

## 7. Discussion

As detailed above, the *OVERGAP* algorithm detects and corrects anomalies during the acquisition of consecutive images. The *OVERGAP* algorithm is also useful to evaluate the performance of the acquisition system over time.

The acquisition of an image every IW/2 m as well as the use of *DTW* and *WD* distances to detect and correct anomalies are the most original aspects of the algorithm. *DTW* distance has been found to be useful here for obtaining distances between columns of different images. *OVERGAP* detects anomalies using *DTW*. The *WD* determines the difference in scale between columns with the same information. *OVERGAP* uses *WD* to correct anomalies by resizing an image.

The examples of anomalies have been simulated. The good performance of *OVERGAP* in detecting and correcting anomalies does not depend on whether the camera position have been simulated or measured with a real device. For this reason and for the sake of simplicity, a *DGPS* have not been used.

An object like the image of scissors has been positive for presenting the results. With this image, it can be observed that there are no significant differences between the image of the initial object and the final image.

We have considered using a metric that can quantify the quality of the image of the scissors (or others) once the anomaly has been corrected. For example, comparing the result with the initial synthetic image with the help of a metric such as Mean Square Error or another similar one. The problem is that the image without anomaly can be cropped, as shown in Figure 9. This result is due to the position of the camera and not *OVERGAP*. Therefore, in this case, a metric could not quantitatively correctly assess the detection and correction of the anomaly. Therefore, we only show images such as those in Figure 8 and Figure 9 to visually evaluate the result.

Multiple identical columns scattered in an image can make it difficult for *OVERGAP* to perform. Indeed, *OVERGAP* searches with *DTW* distance for the column in an image which is most similar to a specific column in another image. The algorithm may fail to detect a potential anomaly if several columns are identical. However, in the context of precision agriculture, images of a row of plants are mainly composed of leaves, branches and fruits and, therefore, it is very difficult to find identical columns. In a similar way, the columns are similar within the area in the scissors image where anomalies are detected, but are not 100% identical. This image allows us to emphasize how *DTW* is able to detect anomalies in the presence of multiple similar, but non-identical, columns.

In addition, *OVERGAP* always requires that the anomaly area is included in the corresponding even image.

The *OVERGAP* algorithm has also been tested to detect and correct simulated anomalies using images of a vineyard. Two solutions have been explained. The first uses a strip with drawings. The behavior of *OVERGAP* is the same as that with the image of the scissors, since it works with the part of the image that contains the strip. The second solution works with sub-images that present a small number of leaves. The images without anomalies for either of the two solutions cannot present perfect continuity, mainly due to the different orientation of the leaves and the irregularity of the ground where the mobile vector moves. However, these images are perfectly valid to be used in a learning algorithm.

For simplicity, gray images have been used. However, *OVERGAP* can also work with color images. From a color image, a gray image can be derived. Using this gray image, the type of anomaly can be detected and corrected. This result is then applied to each of the *R*, *G* and *B* components of the color image. The anomaly can also be detected and corrected using one of the three components. In this case, the result is applied to the other two components.

The most important part of a machine learning project *is preparing the data.* The data must be of quality and representative of the whole. The main result of *OVERGAP* is to obtain quality images where there is no repeated information or lack of information between consecutive images.

Regarding image acquisition in the precision agriculture domain, several methods have been tested to detect and correct anomalies using vineyard images. The best way to detect and correct anomalies is to use *OVERGAP* in conjunction with a drawing strip. Without the strip, the best results have been obtained with *OVERGAP* and *BRISK* for the overlap anomaly and only *OVERGAP* for the gap anomaly. With or without the strip, *OVERGAP* has the advantage of being able to work with a part of the image. When *OVERGAP* works with a strip, the scale is used to correct possible changes in the camera position. Without the strip, it may not be advisable to use scaling (e.g., when the ground is uneven). Unlike *OVERGAP*, the other stitching methods work with all the information in each image and need to detect a minimum number of matched features.

In the future, *OVERGAP* will be integrated into the processor embedded on a mobile vector that is also capable of obtaining radar images [24]. In this way, radar images and images in the visible domain will be obtained simultaneously and data fusion algorithms could be advangeously applied [25]. The information from both types of images may be complementary and their fusion would be positive to derive an efficient prediction model using Machine Learning techniques. With this integration, it will also be possible to determine the maximum speed at which the mobile vector can move to obtain consecutive images in real-time. If the mobile vector is required to move at a higher speed, the *OVERGAP* algorithm can also be used offline.

## 8. Conclusions

*OVERGAP* is a new algorithm for the detection and correction of anomalies during consecutive image acquisition. These images can mainly be used to build a prediction model.

*OVERGAP* is especially useful in the field of precision agriculture. Future work will consider applying *OVERGAP* to vineyard yield prediction.

## Data Availability

Dataset available on request from the authors.
